# Importance of finding the bona fide target of the Fanconi anemia pathway

**DOI:** 10.1186/s41021-019-0122-y

**Published:** 2019-03-06

**Authors:** Wataru Sakai, Kaoru Sugasawa

**Affiliations:** Biosignal Research Center, and Graduate School of Science, Kobe University, 1-1 Rokkodai, Nada, Kobe, Hyogo 657-8501 Japan

**Keywords:** Fanconi anemia, Aldehyde, DNA damage, DNA repair

## Abstract

Fanconi anemia (FA) is a rare genetic disease characterized by the deficiency of the cellular response and repair pathway for DNA interstrand crosslink (ICL) damage. Although recent studies have revealed the detailed molecular functions of FA proteins encoded by 22 genes, the mechanism of occurrence of endogenous ICLs in the human body remains poorly understood. In this short review, we summarize the potential endogenous sources of ICLs counteracted by FA proteins, and provide perspectives on the unanswered questions regarding FA.

## Introduction

**Fig. 1:**
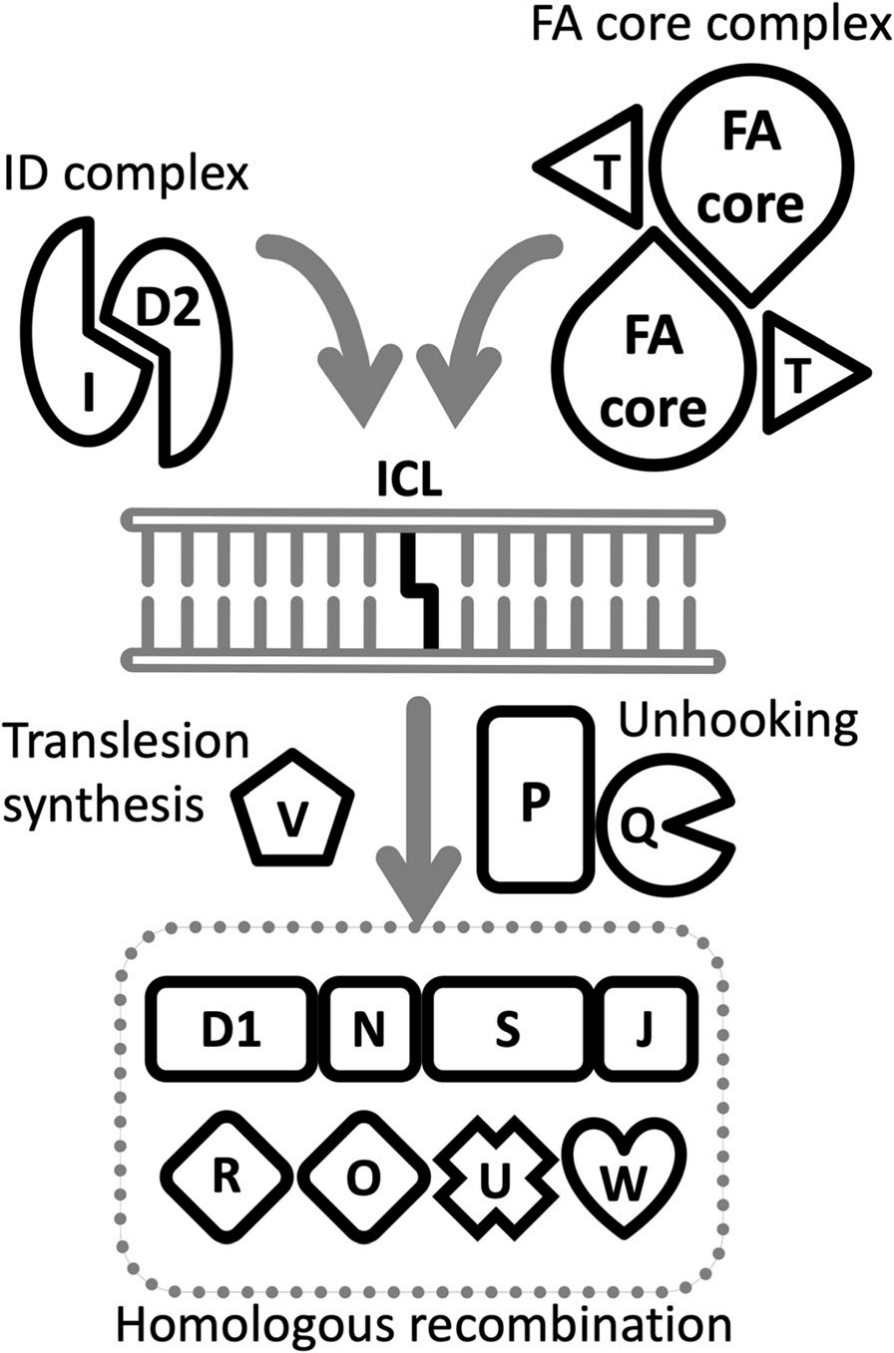
A model for the canonical FA pathway. The FA core complex is a multiple protein complex which contains a homo-dimeric module for ubiquitination. FANCT acts as a ubiquitin-conjugating enzyme associated with the FA core complex. FANCD2 and FANCI form a heterodimer (ID complex), and are known as targets for mono-ubiquitination mediated by the FA core complex. FANCP and FANCQ are involved in DNA strand incisions on either side of the ICL (a process called “unhooking”). FANCV is an accessory subunit of DNA polymerase ζ involved in translesion synthesis over the unhooked ICL. At the final step of ICL repair, a homologous recombination-mediated process restores the fidelity of the genome. For more detailed mechanisms of the canonical FA pathway, see a review in [34]

Fanconi anemia (FA) is a genetically and phenotypically heterogeneous recessive disease associated with congenital abnormalities, bone marrow failure (BMF), and a predisposition to both hematologic malignancies and solid tumors. Moreover, endocrine abnormalities, such as dyslipidemia and metabolic syndrome are common in patients with FA [1]. At present, 22 genes responsible for FA have been identified, and all the encoded proteins function in an intracellular signaling pathway, designated as the FA pathway, which regulates the response to and repair of DNA interstrand crosslinks (ICLs) (Table 1, and Fig. 1). Therefore, at the cellular level, FA is characterized by hypersensitivity to ICLs induced by chemical agents, such as mitomycin C and cisplatin [2]. Bone marrow transplantation is the only known cure for FA-associated hematologic malignancies; however, safe and effective therapies for treating or preventing the increased risk of solid tumors have not yet been established. The two longstanding questions in FA research are, “What are the natural causes of ICLs?” and “What is the origin of these causes?” Even in the absence of environmental exposure to ICL-inducing agents, patients with FA display clinical symptoms at birth or in early life. Detailed mechanisms of these symptoms remain unclear; however, recent studies have revealed important insights into the pathogenesis of FA (see below).

**Table 1:** The FA gene products and their functions

FA protein (alias)	Function
FANCA	FA core complex
FANCB	FA core complex
FANCC	FA core complex
FANCD1 (BRCA2)	Homologous recombination
FANCD2	ID complex
FANCE	FA core complex
FANCF	FA core complex
FANCG	FA core complex
FANCI	ID complex
FANCJ (BRIP1)	Homologous recombination
FANCL	FA core complex, ubiquitin ligase
FANCM	FA core complex, helicase
FANCN (PALB2)	Homologous recombination
FANCO (RAD51C)	Homologous recombination
FANCP (SLX4)	ICL unhooking
FANCQ (ERCC4/XPF)	ICL unhooking, structure-specific endonuclease
FANCR (RAD51)	Homologous recombination
FANCS (BRCA1)	Homologous recombination
FANCT (UBE2T)	Ubiquitin-conjugating enzyme
FANCU (XRCC2)	Homologous recombination
FANCV (MAD2L2/REV7)	Translesion synthesis
FANCW (RFWD3)	Homologous recombination, ubiquitin ligase

## Acetaldehyde and formaldehyde

A wide variety of reactive aldehydes are ubiquitously found in the environment. Acetaldehydes are the major by-products of the catabolic metabolism of alcoholic beverages [3]. Formaldehyde is not only a chemical compound present in some cosmetics and foods, but also a naturally-occurring compound in the human body [4, 5]. The International Agency for Research on Cancer categorizes these aldehydes into “Group 1,” the highest risk factors for carcinogenicity. Recently, an understanding of the effect of these small aldehydes has yielded significant progress on our view of the pathogenesis of FA. Aldehyde dehydrogenase 2 (ALDH2) and alcohol dehydrogenase 5 (ADH5) play important roles in acetaldehyde and formaldehyde detoxification, respectively. Both enzymes oxidize aldehydes, thereby reducing its genotoxic effects. Interestingly, human and chicken FA-deficient cells have been shown to be hypersensitive to plasma levels of formaldehyde without any defect on both ALDH2 and ADH5 [6]. Mice deficient in *Aldh2* displayed chromosomal aberrations and increased mutagenesis in their hematopoietic stem cells [7]. Moreover, combined inactivation of FA genes and *Aldh2* or *Adh5* demonstrated developmental defects, BMF, and a predisposition to leukemia [8–11]. About 540 million of the world’s population, particularly the East Asians, carry a dominant-negative allele (rs671) of *ALDH2* [12]. Alcohol consumption by the individuals with this variant is strongly associated with higher risks of esophageal and rectal cancer [13, 14]. Remarkably, the homozygosity of the *ALDH2* variant in Japanese patients with FA is associated with accelerated progression of BMF compared to the heterozygotes [15]. All these results strongly suggest that the level of endogenous aldehydes is important for the pathogenesis of FA.

## Lipid peroxidation-derived aldehydes

Lipids are crucial cellular membrane components, as well as essential energy sources in the human body; however, lipid metabolism and peroxidation produce a variety of aldehydes, such as 4-hydroxynonenal (4HNE) and malondialdehyde (MDA) [16]. These aldehydes are abundant in the human body, and react with proteins and DNA to form biomolecule adducts associated with carcinogenesis and neurodegenerative diseases [16, 17]. MDA is one of the most predominant products of lipid peroxidation, and is generated primarily by decomposition of poly-unsaturated fatty acids with at least two methylene-interrupted double bonds [17]. Although it has been proposed that MDA could form ICLs and is mutagenic in human cells [18], the chemical reactivity of MDA is not high under physiological conditions [19]. Conversely, MDA has a unique ability to form hybrid products with acetaldehyde and formaldehyde. Notably, these “hybrid aldehydes” can react with nucleosides and amino acids under physiological conditions, indicating their ability to form ICLs or protein adducts [20–22]. Some studies have reported intriguing implications of lipid metabolism in FA. Endocrine abnormalities including dyslipidemia, obesity, and metabolic syndrome are present in more than 70% of FA patients [23]. At the cellular level, metabolome analysis of mesenchymal stromal cells from FA-knockout mice revealed abnormal lipid profiles, especially in glycerophospholipid biosynthesis [24]. FA-deficient human cells also show accumulation of lipid droplets (LDs) [25], which are nuclear and cytoplasmic organelles that store neutral lipids and are important for energy metabolism. Recently, it has been suggested that LDs may serve as a biomarker for metabolic diseases [26]. Consistent with these results, lipidomic profiling in FA-deficient human keratinocytes also showed upregulation of glycosphingolipids [27]. Further analyses are required to elucidate whether these lipid-related dysregulations in FA depend on the secondary effects of the deficiency in the canonical FA pathway or they are the direct effects of the unknown function(s) of FA proteins.

## Conclusions

An understanding of natural ICLs may play an important role in the development of an effective treatment for FA. Avoiding exposure to endogenous ICL sources or reducing the occurrence of endogenous ICLs may suppress the onset of FA pathogenesis. Indeed, avoidance of sun-exposure is an effective treatment for patients with xeroderma pigmentosum, which is associated with a deficiency of nucleotide excision repair that removes DNA lesions induced by sunlight (ultraviolet radiation) [28]. Alternatively, an understanding of natural ICLs has allowed the development of molecular-targeted drugs, such as alda-1 (ALDH2 agonist) and metformin (aldehyde scavenger) [29, 30], for the prevention of cancer or BMF in patients with FA.

Over the last two decades, researchers have made great progress in elucidating the molecular mechanisms involving FA proteins. The functional integrity of FA proteins is also important for acquired resistance to anticancer drugs [31–33]. However, there are still some questions to be answered in FA research. Finding the bona fide target of the FA pathway would not only contribute to the alleviation of FA symptoms, but also improve the quality of life of humans in general.

## Abbreviations

ADH: Alcohol dehydrogenase
ALDH: Aldehyde dehydrogenase
BMF: Bone marrow failure
FA: Fanconi anemia
ICL: Interstrand crosslink
MDA: Malondialdehyde
